# Testing Silica Fume-Based Concrete Composites under Chemical and Microbiological Sulfate Attacks

**DOI:** 10.3390/ma9050324

**Published:** 2016-04-29

**Authors:** Adriana Estokova, Martina Kovalcikova, Alena Luptakova, Maria Prascakova

**Affiliations:** 1 Institute of Environmental Engineering, Faculty of Civil Engineering, Technical University of Kosice, Kosice 04200, Slovak Republic; martina.kovalcikova@tuke.sk; 2 Institute of Geotechnics, Slovak Academy of Sciences, Kosice 04001, Slovak Republic; luptakal@saske.sk (A.L.); prascak@saske.sk (M.P.)

**Keywords:** concrete, corrosion, leaching, silica fume

## Abstract

Current design practices based on descriptive approaches to concrete specification may not be appropriate for the management of aggressive environments. In this study, the durability of cement-based materials with and without the addition of silica fume, subjected to conditions that leach calcium and silicon, were investigated. Chemical corrosion was simulated by employing various H_2_SO_4_ and MgSO_4_ solutions, and biological corrosion was simulated using *Acidithiobacillus* sp. bacterial inoculation, leading to disrupted and damaged surfaces; the samples’ mass changes were studied following both chemical and biological attacks. Different leaching trends were observed via X-ray fluorescence when comparing chemical with biological leaching. Lower leaching rates were found for concrete samples fortified with silica fume than those without silica fume. X-ray diffraction and scanning electron microscopy confirmed a massive sulfate precipitate formation on the concrete surface due to bacterial exposure.

## 1. Introduction

Concrete structures are generally considered to be durable because of their longer service life; however, they can deteriorate for a variety of reasons, including material limitations, poor quality design, and construction practices, as well as exposure to extreme environmental conditions [1]. Deterioration will occur when an environmental agent breaks the inorganic bonds of the cement binder. Acids, sulfates, NH_4_^+^ and Mg^2+^ salts, alkalis, organic esters, and CO_2_ can destroy the binder over time [2,3].

Sulfate destruction of concrete occurs as a result of both chemical and physical processes [4,5]. Two main reactions are involved, namely, the reaction of SO_4_^2−^ with hydrated calcium aluminates forming ettringite and the combination of SO_4_^2−^ with free Ca(OH)_2_ forming gypsum [6]. Considerable increases in volume are due to both reactions, causing the expansion and disruption of the hardened concrete. More recently, a third form of sulfate attack, known as thaumasite attack, has been discovered. Thaumasite is a calcium silicate sulfate-carbonate hydrate (Ca_3_Si(OH)_6_(CO_3_)(SO_4_)·12H_2_O) that forms at temperatures below 15 °C by a reaction between cement paste hydrates, carbonates, and sulfate ions. Its formation reduces the cement paste to a soft mulch [7].

Concrete bio-deterioration is primarily due to the metabolic activity of sulfur-oxidizing bacteria (SOB) belonging to the *Acidithiobacillus* and *Thiobacillus* genera, *A. thiooxidans*, and sulfate-reducing bacteria (SRB) [8]. SRB metabolic activity leads to the production of H_2_S, which is oxidized by SOB to H_2_SO_4_. Biogenic H_2_S is the metabolic end product of sulfate-reducing bacteria living in anaerobic zones of soil, polluted water, and sewage. The presence of H_2_S and H_2_SO_4_ usually induces the corrosion of concrete sewer pipes that are inadequately ventilated and have low sewage flow [9,10]. The concentration of biogenic H_2_SO_4_ in concrete pore solutions may be as high as 10%, corresponding to a pH value of less than 1.0. In some cases, the pore solution has reached a pH as low as 0.6 because of microbiologically induced attacks, even at an initial alkalinity of pH 13 [11].

Supplementary cementitious materials, such as silica fume, ground granulated blast-furnace slag, and natural pozzolans, are added to concrete as part of the total cementitious system to improve the durability of concrete in an aggressive sulfate environment [12,13,14,15]. The addition of silica fume is assumed to improve the durability of concrete via a reduction in permeability, which refines pore structure, leading to the suppression of the diffusion of harmful ions, reducing Ca(OH)_2_ content and, in turn, increasing resistance to sulfate attack [16,17,18,19]. Additionally, silica fume has been recognized as a pozzolanic admixture that is effective in enhancing mechanical properties to a great extent [20,21,22]. Silica fume, a mineral admixture composed of very fine solid glassy spheres of SiO_2_, is a by-product of the smelting processes in the silicon and ferrosilicon industry [23]. Silica fume particles are extremely small, with more than 95% of the particles finer than 1 μm and generally 50- to 100-fold finer than average cement or fly ash particles [24]. When used in conjunction with Portland or blended cement, silica fume improves the microstructure of concrete by means of a microfilling ability and pozzolanic activity. During this process, waste is also transformed to reusable materials to help reduce industrial waste, thereby facilitating sustainable construction [25].

This study presents the results of different methods of analyzing the deterioration of silica-fume-based concrete due to the metabolic activity of sulfur-oxidizing bacteria and simulates an acidic and sulfate chemical corrosion. Various studies have focused on the investigation of silica fume-based concrete subjected only to chemical sulfate attack [26,27,28]. The significance of this research study lies in a comparison of both chemical and microbiological sulfate exposure, including the effect of the silica fume admixture used to improve concrete quality.

The objective of this study was, using X-ray fluorescence spectrometry (XRF), to examine the resistance of concrete composites with the addition of silica fume when considering the leaching of the primary cement matrix components Ca^2+^ and Si^4+^ due to sulfate exposure. The results are also analyzed in the context of the Si^4+^ and Ca^2+^ leaching rates, calculated by considering the maximum measured amount of Ca^2+^ (or Si^4+^) in the leachates, and the mass changes of concrete samples after the experiment due to the formation of surface precipitates using scanning electron microscopy/energy dispersive X-ray microanalysis (scanning electron microscopy/energy dispersive X-ray microanalysis, SEM/EDX) and X-ray powder diffraction (XRD).

## 2. Results and Discussion

The major components of the concrete samples measured via XRF prior to the experiment are illustrated in Table 1 in oxide form.

The M1 samples with the addition of silica fume (5 wt % of total binder mass) contained a higher percentage of SiO_2_ (up to 45.63%) compared with the M0 samples (30.16%) without the addition of silica fume, as can be observed in Table 1. The difference in SiO_2_ content was linked with the chemical composition of silica fume, which was composed of more than 90% SiO_2_ (see Section 3.1). However, the percentage of CaO decreased in silica fume-based samples by 5% compared with samples without silica fume. Only small differences in the concentrations of the other components were detected between the M0 and M1 concrete samples.

### 2.1. Chemical Leaching of Si^4+^ and Ca^2+^

**Figure 1:**
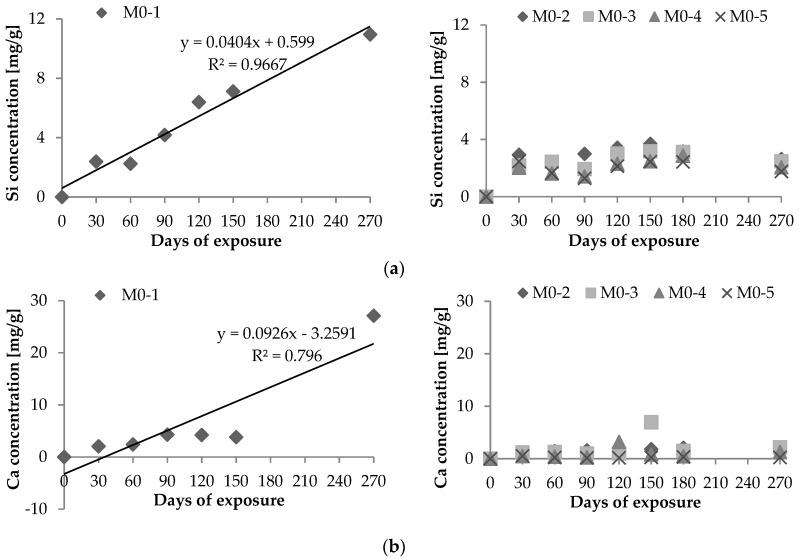
Dissolved: (**a**) Si^4+^ and (**b**) Ca^2+^ corresponding to 1 g of concrete samples without the addition of silica fume.

Different leaching trends were observed for the various media (Figure 1). The leaching of Si^4+^ and Ca^2+^ appears to increase with exposure duration (*R* = 0.98 and *R* = 0.89, respectively) for H_2_SO_4_ at pH 3.0 (M0-1) over 270 days; in other media, less Si was leached out over time. This was also naturally true for Ca^2+^ and is in accordance with the literature [29]. Regarding the media-leaching efficiency, the highest quantities of dissolved Si^4+^ and Ca^2+^ were measured in H_2_SO_4_ with a pH of 3.0, whereas the lowest concentrations were observed in fresh water throughout the experiment.

**Figure 2:**
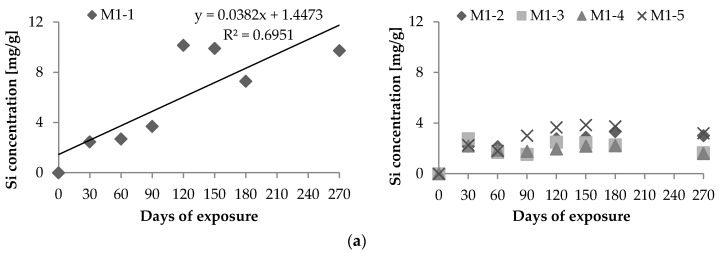

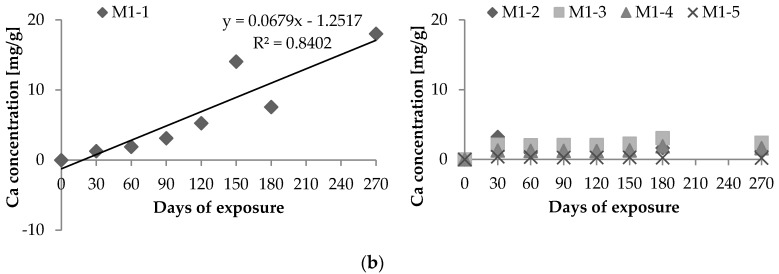
Dissolved: (**a**) Si^4+^ and (**b**) Ca^2+^ corresponding to 1 g of concrete samples with the addition of silica fume.

Si^4+^ and Ca^2+^ leaching trends of concrete samples with silica fume are illustrated in Figure 2 for various sulfate environments as well as fresh water. Quantities of dissolved Si^4+^ and Ca^2+^ correspond to 1 g of concrete samples.

The most intensive leaching of Si^4+^ (10.2 mg·g^−1^) during the 270 days of exposure was observed for concrete sample M1-1 exposed to an aggressive environment of H_2_SO_4_ with a pH of 3 as observed in Figure 2a. The concentration of dissolved Si^4+^ in leachates was the lowest for sample M1-4 immersed in a solution of MgSO_4_ with a concentration of SO_4_^2−^ 3 g·L^−1^ (Figure 2a). Senhadji *et al.* [17] attributed the effect of silica fume on sulfate resistance more to chemical effects than reduced permeability while investigating the resistance of concrete to decomposition in MgSO_4_ and Na_2_SO_4_ solutions. Zelic *et al.* mentioned that a silica fume replacement enhances the durability of mortar exposed to magnesium sulfate attack by lowering the lime content, thereby increasing the initial compressive strength; this occurs due to the pozzolanic reaction [30].

The maximum amount of dissolved Ca^2+^ (18.0 mg·g^−1^) was observed in the leachate of sample M1-1 after 270-day experiments (Figure 2b). The concrete sample M1-5 exposed to fresh water was found to have the lowest values of leached-out Ca^2+^ during the experiment. Similar to concrete samples without silica fume, different leaching courses have been identified in concrete samples with silica fume. H_2_SO_4_ (pH 3) was confirmed to be the most aggressive towards concrete, exhibiting a linear correlation between the leached Si^4+^ and Ca^2+^ concentrations with *R* = 0.83 and 0.92 exposure times, respectively.

**Figure 3:**
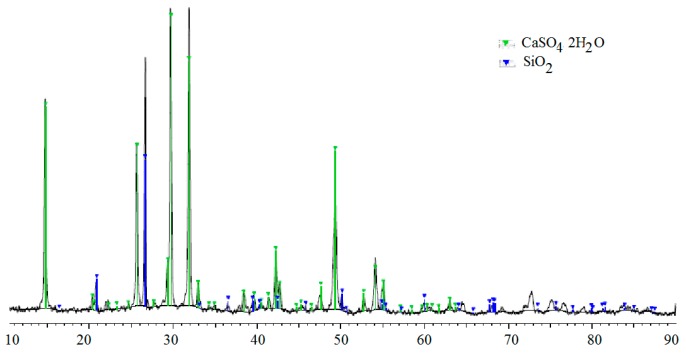
X-ray powder diffraction (XRD) diffractogram of the surface precipitates.

The leaching courses of the other media exhibited an increasing trend until 150 or 180 days of the exposure, after which leaching decreased. The lower concentrations of the Si^4+^ and Ca^2+^ in the leachates at the end of the experiment compared with the maximum at days 150 and 180, respectively, could be explained by the precipitation of newly formed compounds containing Ca^2+^ and Si^4+^ on the concrete surfaces as observed using X-ray powder diffraction (XRD). Traces of gypsum and quartz on the surface of concrete samples were also confirmed (Figure 3).

The similarity between leaching courses of Si^4+^ and Ca^2+^ for both silica fume- and non-silica fume-based concrete samples was confirmed for the media used in the chemical corrosion simulation.

### 2.2. Biological Leaching of Ca^2+^ and Si^4+^

**Figure 4:**
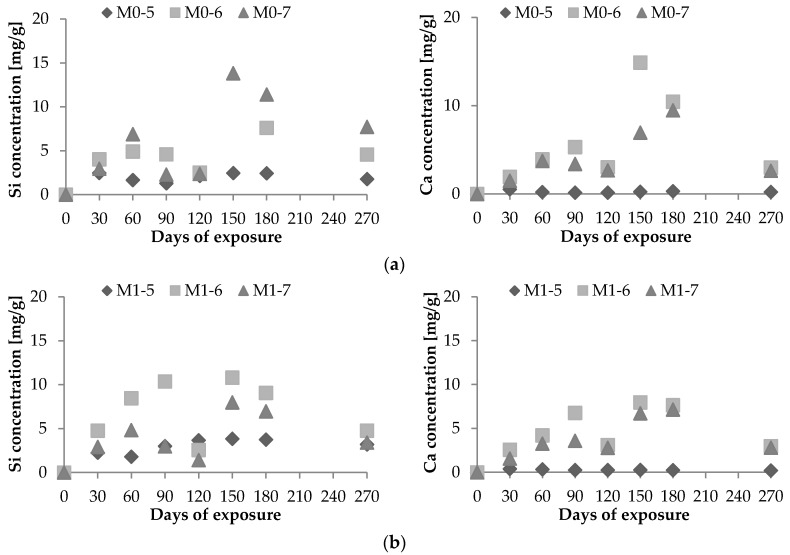
Dissolved Si^4+^ and Ca^2+^ corresponding to 1 g of concrete samples (**a**) without and (**b**) with the addition of silica fume.

Substantially more intensive Si^4+^ and Ca^2+^ leaching was found in the presence of *Acidithioxidans* bacteria (M0-6 and M0-7 samples) compared with fresh water (M0-5) than previously assumed (Figure 4).

No linear correlation between dissolved concentrations of Si^4+^ and Ca^2+^ and exposure time was observed. With the exception of the concentrations measured on day 120, it can be concluded that the concentrations of dissolved Si^4+^ and Ca^2+^ increased until day 150 of the bacterial exposure and thereafter significantly decreased. This can be related to the formation of massive sulfate precipitants on the surface of the concrete samples as reported in the study by Nielsen *et al.* [31]. The white covering of the samples under bacterial attack was more intensive compared with concrete samples subjected to chemical attack. By decreasing the lime content in mortars during Mg-sulfate immersion, the formation of gypsum and ettringite, which are responsible for decreasing mortar durability, decreases [32].

### 2.3. Comparison of Chemical and Biological Corrosion

**Figure 5:**
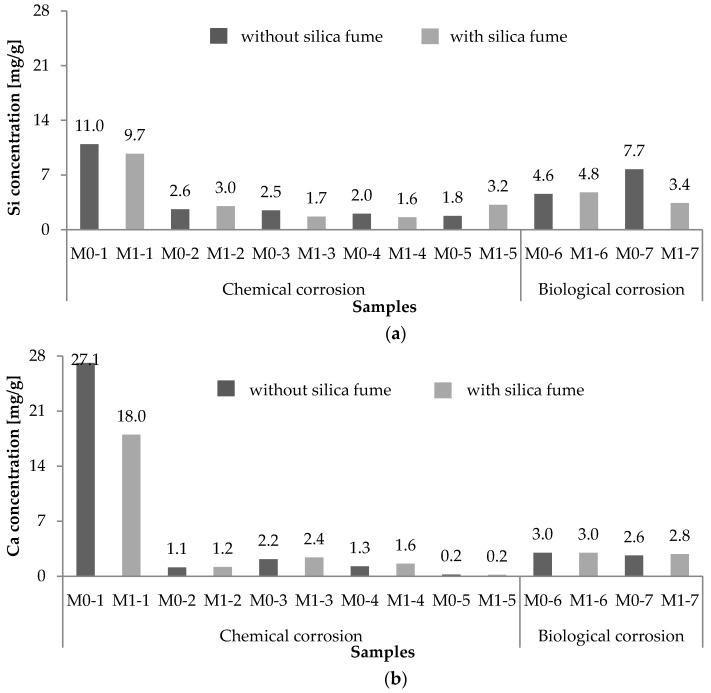
Leached-out masses of (**a**) Si and (**b**) Ca after the 270-day experiment.

Figure 5 shows a comparison of quantities of leached Si^4+^ and Ca^2+^ due to both chemical and biological corrosion from samples made of two different mixtures after 270-day experiments corresponding to a 1-g concrete sample.

By comparing two different mixtures of cement composites made of ordinary Portland cement without silica fume (M0) and with silica fume (M1), the concrete mixture with silica fume was found to be more durable, in terms of Si^4+^ leaching, when exposed to aggressive environments of H_2_SO_4_ with a pH of 3, both MgSO_4_ solutions, and a diluted bacterial medium. However, lower durability, after the evaluation of Si^4+^ leaching, was detected in the H_2_SO_4_ with a pH of 4.0, concentrated bacterial medium, and fresh water.

When comparing the chemical and biological corrosion (Figure 5a), Si^4+^ leaching was more significant when subjected to bacterial exposure, with the exception of H_2_SO_4_ with a pH of 3 (M0-1 and M1-1 samples).

As for Ca^2+^ leaching, the concrete mixture with silica fume was found to be more durable in the aggressive environment of H_2_SO_4_ with a pH of 3, concentrated bacterial medium, and fresh water than in the other aggressive environments, as can be observed in Figure 5b. Based on the leached-out masses of Ca^2+^ after the experiment, bacterial exposure was found to be the most significant compared with the chemical exposure with the exception of H_2_SO_4_ with a pH of 3. However, the Si^4+^ and Ca^2+^ concentrations at the end of the 270-day experiment likely do not represent the total amounts of dissolved ions. Therefore, the Si^4+^ and Ca^2+^ leaching rates were calculated by considering the maximum measured amount of Ca^2+^ (or Si^4+^) in the leachates. The leaching rate *V_d_* was calculated by dividing the measured mass of Si^4+^ or Ca^2+^ in a particular aggressive environment according to the corresponding time of exposure, as shown in Equation (1), based on the work of Ikeda *et al.* [33]:

(1) $$V_{d}=\frac{X_{d}}{T\times S}$$

where

*V_d_*: Si^4+^ (or Ca^2+^) leaching rate per unit area (μg·h^−1^·cm^−2^);
  *X_d_*: maximum amount of Si^4+^ (or Ca^2+^) leached out during the experiment (μg);
  *T*: period of test [=24 × days of leaching (hours)]; and
  *S*: area of exposure surface (cm^2^).

Calculated Si^4+^ and Ca^2+^ leaching rates are reported in Table 2.

Lower leaching rates have been identified for concrete samples with the addition of silica fume (M1) as opposed to samples without silica fume (M0) in corresponding media (Table 2). As reported by Lee *et al.*, the incorporation of 10% silica fume in ordinary Portland cement matrix showed that the total reduction in strength was greater for mortar specimens without silica fume compared with those with silica fume [26]. Similarly, Ganjian and Pouya discovered that the performance of pastes and concrete specimens with silica fume exposed to simulation ponds and a site tidal zone were inferior to those without the silica fume replacement [34]. However, Hekal *et al.* reported that a partial replacement of Portland cement by silica fume (10%–15%) did not show a significant improvement in sulfate resistance of hardened cement pastes [35].

Higher rates of Si^4+^ than Ca^2+^ leaching were detected for all samples subjected to bacterial attack. A comparison of the Si^4+^ and Ca^2+^ leaching rates due to H_2_SO_4_ (pH 4) attack and a bacterial medium with the same pH of 4 revealed that the bacterial attack was more aggressive.

To confirm the superior performance of silica fume-based concrete in an aggressive sulfate environment, the mass percentage of the dissolved ions (Table 3) was also calculated. The percentage of dissolved ions was calculated by dividing the maximum quantity of dissolved ions by the total quantity of ions in concrete samples prior to the experiment.

The superior performance of concrete samples based on silica fume in terms of both Si^4+^ and Ca^2+^ leachability was confirmed for all concrete samples with the exception of samples immersed in fresh water. The most significant difference was noticed for samples subjected to bacterial attack. The calculated leachable fraction of Si^4+^ was 5.2- and 3.3-fold higher for samples without silica fume compared with the samples with silica fume after bacterial inoculation. Significantly lower leachable fractions of both Si^4+^ and Ca^2+^ ions were also observed for silica fume-based samples exposed to H_2_SO_4_ with a pH of 3 (2.9-fold for Si^4+^ and 2.3-fold for Ca^2+^).

The results of the mass changes of the analyzed concrete samples prior to and after the experiments are given in Table 4. 

A decrease in mass was noticed for all concrete specimens after the chemical corrosion experiments, whereas an increase in mass was detected for all samples after the biological corrosion experiments, as can be observed in Table 4. The increase in mass for all samples under bacterial exposure is likely a result of the formation of massive precipitants on the surfaces of the concrete.

**Figure 6:**
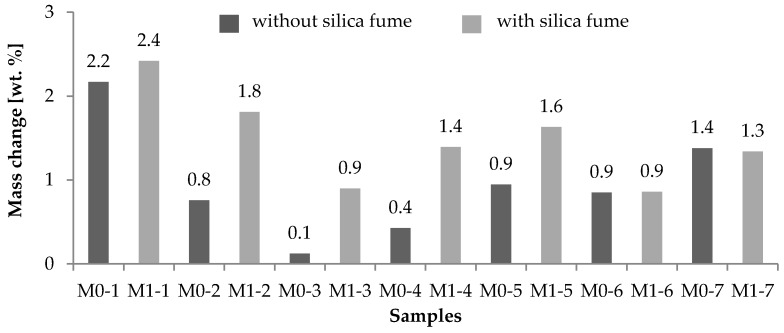
Mass changes of concrete samples.

A histogram of mass changes of the concrete specimens with and without the addition of silica fume prior to leaching and after 270 days of leaching is shown in Figure 6.

The percentage of mass changes for concrete samples varied from 0.12% (sample M0-3) to 2.42% (sample M1-1). The highest decrease in concrete mass was detected for samples exposed to the most aggressive environment, represented by H_2_SO_4_ with a pH of 3, which corresponds with the findings regarding the leaching of Si^4+^ and Ca^2+^. Surprisingly, higher mass changes were found for all concrete samples with the addition of silica fume than samples without silica fume. As is known, the deterioration of concrete can be caused by both mechanisms: (i) a dissolution of the cement paste constituents and its subsequent removal from the paste matrix due to its inherently high solubility and (ii) chemical reactions within the paste, e.g., salt crystallization, resulting in concrete volume expansion. The decrease in mass is linked with the leaching process, whereas an increase in mass can be linked with the penetration of sulfate solutions either by simple diffusion or capillary suction, which causes some salts to undergo cycles of dissolution and crystallization. The mass changes after chemical exposure indicate that the leaching process dominates in silica fume-based samples, whereas the crystallization process dominates in concrete samples without silica fume. 

**Figure 7:**
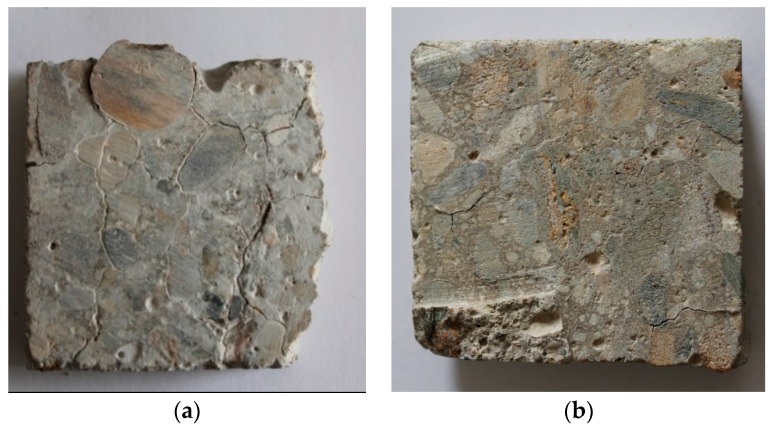
Samples (**a**) without and (**b**) with the addition of silica fume after the chemical exposure.

**Figure 8:**
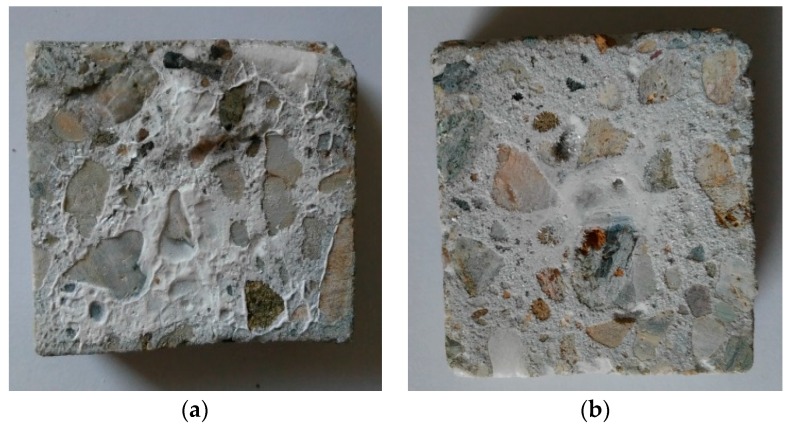
Samples (**a**) without and (**b**) with the addition of silica fume after the bacterial exposure.

The formation of easily visible corrosion-induced cracks on the surface of concrete samples exposed to different aggressive media has been observed (Figure 7 and Figure 8). No significant changes were identified in concrete samples M0-5 or M1-5 immersed in fresh water with a pH of 7.2. 

**Figure 9:**
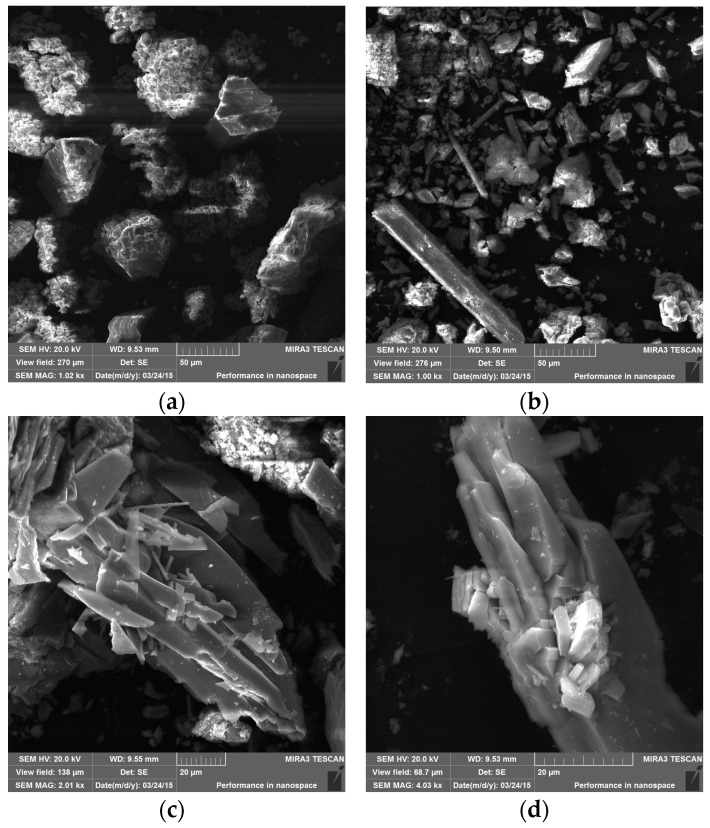
Scanning electron microscopy (SEM) micrographs of (**a**) M0-2; (**b**) M1-2; (**c**) M0-6; and (**d**) M1-6 samples.

**Figure 10:**
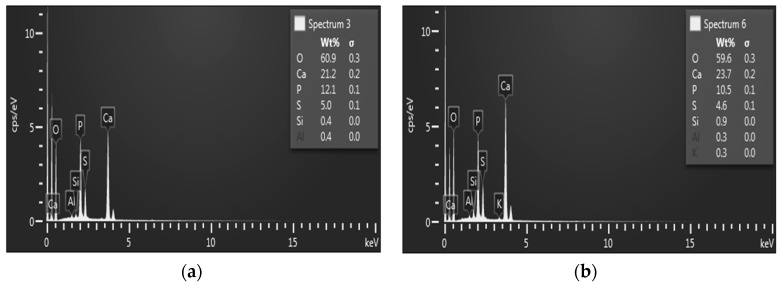

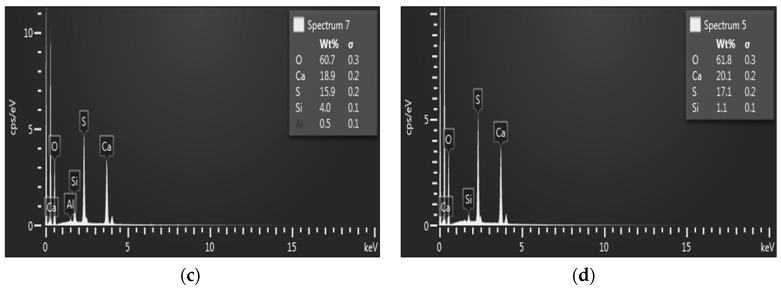
Energy dispersive X-ray microanalysis (EDX) semi-quantitative analysis of (**a**) M0-6; (**b**) M1-6; (**c**) M0-2; and (**d**) M1-2 samples.

The surfaces of the concrete samples under chemical exposure contained only traces of precipitates, whereas the surface of the concrete samples under bacterial exposure was nearly completely covered by white crystalline compounds. The concrete samples with and without silica fume exposed to H_2_SO_4_ with a pH of 4 (M0-2 and M1-2 samples) and concentrated bacterial medium with a pH of 4 (M0-6 and M1-6 samples) were also analyzed using SEM and EDX, similar to our previous work [36]. The presence of the new surface products containing SO_4_^2−^ (gypsum and thaumasite) was observed via SEM (Figure 9) and EDX (Figure 10).

The surface products observed were analyzed by EDX to confirm the presence of Ca^2+^ and Si^4+^ compounds (Figure 10).

As can be observed in Figure 10c,d, the presence of Ca, Si, O, and S in the surface compounds was confirmed. Based on the EDX and XRD analyses presented above, the presence of thaumasite (Ca_3_Si(OH)_6_(CO_3_)(SO_4_)·12H_2_O) and gypsum (CaSO_4_·2H_2_O) can be assumed to be acting on the concrete surfaces. As reported by Schmidt *et al*. [37], despite the fact that thaumasite is thermodynamically favorable and more stable at lower temperatures, it can also be detected at 20 °C at low concentrations after sulfate interaction. Secondary gypsum forms parallel to thaumasite at high concentrations of SO_4_^2−^.

## 3. Materials and Methods

### 3.1. Cement and Silica Fume

The concrete samples used in the experiment were prepared using cement CEM I 42.5 N (Povazska cementaren, Ladce, Slovak Republic). The basic chemical composition of cement measured using XRF (SPECTRO iQ II, Spectro-Ametek, Kleve, Germany) in wt % was as follows: 1.37 MgO, 4.02 Al_2_O_3_, 18.11 SiO_2_, 0.33 P_2_O_5_, 1.49 SO_3_, 0.06 Cl, 1.12 K_2_O, 57.15 CaO, 0.18 TiO_2_, 0.001 MnO, and 2.70 Fe_2_O_3_. Basic components of silica fume (OFZ a.s., Istebne, Slovakia), representing a mineral waste admixture, measured using XRF in wt % were as follows: 1.07 MgO, 0.36 Al_2_O_3_, 92.46 SiO_2_, 0.05 P_2_O_5_, 0.07 SO_3_, 0.14 Cl, 0.99 K_2_O, 0.23 CaO, 0.58 MnO, and 1.24 Fe_2_O_3_.

### 3.2. Concrete Composite Samples

Two mixtures (M0 and M1), per m^3^, consisting of cement (Povazska cementaren), silica fume (OFZ a.s.), water, aggregate (Vychodoslovenske stavebne hmoty, Geca, Slovakia) with particle size fractions of 0/4 mm, 4/8 mm, and 8/16 mm, and plasticizer Stachement 2353 based on polycarboxylates (Stachema, Bratislava, Slovakia) were employed (Table 5). According to the literature, the water demand of concrete containing silica fume increases with increasing amounts of silica fume.

Mixtures M0 and M1 were designed to meet the requirement of at least C30/37 concrete strength class and a water-cement ratio (w/c) of a maximum of 0.5 in accordance with EN 206-1. The concrete samples met the criteria for exposure class XA2 [38] with water to cement ratios of 0.47 (M0) and 0.49 (M1) and compressive strengths of 41.2 (M0) and 46.9 MPa (M1); their bulk densities were 2350 (M0) and 2380 kg·m^−3^ (M1). Slightly different water to cement ratios of M0 and M1 concrete mixtures were designed to maintain consistency (Slump S2) in the mixtures. Standardized concrete prisms measuring 100 × 100 × 400 mm^3^ were poured and cured for 28 days immersed in water at the ambient temperature of 20 °C prior to testing [39,40]. The prisms were cut into smaller prisms measuring 50 × 50 × 10 mm^3^ for corrosion testing. They were slightly brushed to remove loose particles, sterilized in 70% ethanol for 24 h to disinfect the concrete surfaces before the bacterial experiment, and dried at 105 °C until a constant mass was reached. Constant mass was achieved when less than 0.1% of the test sample wet mass is lost during additional exposure to the drying process.

### 3.3. Chemical Corrosion Simulation

The latter concrete prisms were exposed by immersion into 400 mL of 0.005 wt % H_2_SO_4_, pH 3.0 (samples: M0-1, M1-1), and 0.0005 wt % H_2_SO_4_, pH 4.0 (samples: M0-2, M1-2), in glass beakers, both being daily checked for pH (FG2-FiveGo, Mettler-Toledo, Switzerland) and kept constant by the addition of 0.1 M H_2_SO_4_. Other prisms were immersed into 400 mL of MgSO_4_ solution of 12.5 g·L^−1^ with SO_4_^2−^ 10 g·L^−1^ (samples M0-3 and M1-3), an MgSO_4_ solution of 3.75 g·L^−1^ with SO_4_^2−^ 3 g·L^−1^ (samples M0-4 and M1-4), and fresh water with a pH of 7.2 (samples M0-5 and M1-5) for concrete corrosion resistance testing [38]. The volume of the liquid media was based on the volume ratio of the concrete prisms and liquid phase (S/L) 1:10. Leaching durations were set at intervals of 30, 60, 90, 120, 150, 180, and 270 days at a temperature of 23 °C. After each 30-day period, the pH and the concentrations of the dissolved Ca^2+^ and Si^4+^ were measured in leaching media.

### 3.4. Microbial Corrosion Simulation

A culture of *A. thiooxidans* was isolated from an acid mine drainage (Pech shaft in the locality of Smolnik, Eastern Slovakia) as described by Waksman and Joffe [41].

The concrete prisms were exposed at 23 °C in covered glass jars to 400 mL of a bacterial culture medium consisting of 20 vol. % of bacterial inoculum and 80 vol % nutrient medium with pH 4.0 according to Waksman and Joffe (samples M0-6 and M1-6) or the same medium diluted with distilled water 1:1 (samples M0-7 and M1-7). Six milliliters of bacterial culture was inoculated at 7-day intervals over a period of 270 days. The concentrations of dissolved Si^4+^ and Ca^2+^ were measured in the leachates every 30 days.

### 3.5. Analytical Methods

The concentrations of Ca and Si in the leachates were determined using XRF (SPECTRO iQ II, Spectro-Ametek, Kleve, Germany) and a silicon drift detector with a resolution of 145 eV at 10,000 pulses. The primary beam was polarized with a Bragg crystal and highly ordered pyrolytic graphite target. Measurements were performed for 300 and 180 s at voltages of 25 kV and 50 kV and currents of 0.5 and 1.0 mA in the presence of helium.

To determine the chemical composition prior to and after the experiments, cement composites were crushed into powder (MSK-SFM-1 Desk-Top planetary ball mill, MTI Corporation, Richmond, VA, USA), and 5 g was mixed with 1 g of a dilution material (Hoechst wax C micropowder, Merck Millipore, Darmstadt, Germany) and subsequently pressed into a pellet at a pressure of 0.1 MPa·m^−2^; this was analyzed using XRF.

The precipitates on the surface of the concrete samples after the experiments were removed and placed on adhesive carbon tape and studied using SEM/EDX (MIRA3 FE, Tescan, Brno, Czech Republic/Oxford Instruments, Abingdon, UK) and XRD (D2 PHASER diffractometer, Bruker, Germany) with Cu K_α_ radiation generated at 10 mA and 30 kV with a step size of 0.04° over the range 2θ from 10° to 90°.

## 4. Conclusions

This paper presents the results of a study on the deterioration of concrete samples caused by chemical and microbiological attacks. Attention was paid to the leachability of the most important components of the cement matrix: Si(IV) and Ca(II) compounds. The mass and surface changes due to this deterioration were of interest as well. The following conclusions have been obtained:

1. Microbiological attack precipitated by *Acidithiobacillus thiooxidans* was more dangerous, in terms of leaching of the main components, than chemical sulfate attack with the exception of a H_2_SO_4_ solution of a pH of 3.
2. The concrete samples equipped with silica fume exhibited better durability, in terms of the leaching of Si^4+^ and Ca^2+^, than concrete samples without silica fume.
3. Significantly lower leachable fractions, specifically those 2.9- and 2.3-fold lower for Si^4+^ and Ca^2+^, respectively, were observed for silica fume-based samples exposed to H_2_SO_4_ with a pH of 3 compared with samples without silica fume.
4. The calculated leachable fraction of Si^4+^ was 5.2- and 3.3-fold higher for samples without silica fume compared with the samples with silica fume under biological exposure.
5. Silica fume concrete is not impervious to all aggressive chemicals. However, the study shows that at a low water to cement ratio (w/c), silica fume concrete can effectively prevent significant damage following many types of chemical attack including sewage leachate. Based on this finding, silica fume concrete has been specified for use in sewer and outfall pipes.

Notably, many problems related to both the chemical and microbial corrosion of concrete have not been solved up to now despite considerable progress in the recognition of corrosion kinetics and mechanisms. Thus, further research on corrosion processes and methods of protection against these processes is necessary.

## Figures and Tables

**Table 1:** Chemical analysis of the analyzed concrete samples.

Mixture	Chemical Composition (wt %)												
	Na_2_O	MgO	Al_2_O_3_	SiO_2_	P_2_O_5_	SO_3_	Cl	K_2_O	CaO	TiO_2_	MnO	Fe_2_O_3_	Other
M0	0.11	3.4	5.21	30.16	0.10	2.89	0.02	0.77	31.27	0.27	0.37	4.4	21.75
M1	0.11	2.73	5.39	45.63	0.09	2.72	0.02	0.79	26.17	0.26	0.36	3.75	11.98

Note: wt. %: weight percentage.

**Table 2:** The leaching rates of Si and Ca.

**Samples without SF (M0)**							
**Element**	**Chemical Corrosion**					**Biological Corrosion**	
	M0-1	M0-2	M0-3	M0-4	M0-5	M0-6	M0-7
*V_d max_* × 10^−2^ μg/(h × cm^2^)							
Si	6.410	1.466	1.259	0.949	4.906	10.537	5.486
Ca	16.942	0.677	2.759	1.584	1.044	5.906	3.134
**Samples with SF (M1)**							
**Element**	**Chemical Corrosion**					**Biological Corrosion**	
	M1-1	M1-2	M1-3	M1-4	M1-5	M1-6	M1-7
*V_d max_* × 10^−2^ μg/(h × cm^2^)							
Si	3.933	1.102	5.504	0.732	1.529	4.288	3.170
Ca	3.969	6.353	1.008	0.620	0.775	3.163	2.367

**Table 3:** The wt % of dissolved Si and Ca.

**Samples without SF (M0)**							
**Element**	**Chemical Corrosion**					**Biological Corrosion**	
	M0-1	M0-2	M0-3	M0-4	M0-5	M0-6	M0-7
Si	13.75	2.61	2.25	2.04	1.76	26.29	31.63
Ca	22.21	0.89	3.02	1.38	0.23	6.45	13.26
**Samples with SF (M1)**							
**Element**	**Chemical Corrosion**					**Biological Corrosion**	
	M1-1	M1-2	M1-3	M1-4	M1-5	M1-6	M1-7
Si	4.76	1.56	1.30	1.04	1.81	5.07	9.60
Ca	9.62	1.71	1.63	1.00	0.21	4.26	9.82

**Table 4:** Changes in mass after the 270-day experiment.

**Samples without SF (M0)**							
**Mass (g)**	**Chemical Corrosion**					**Biological Corrosion**	
	M0-1	M0-2	M0-3	M0-4	M0-5	M0-6	M0-7
Before the experiment	69.61	69.81	73.19	81.96	83.47	76.91	74.73
After the experiment	68.10	69.28	73.10	81.61	82.68	77.56	75.76
Mass change (g)	1.51	0.53	0.09	0.35	0.79	−0.65	−1.03
**Samples with SF (M1)**							
**Mass (g)**	**Chemical Corrosion**					**Biological Corrosion**	
	M1-1	M1-2	M1-3	M1-4	M1-5	M1-6	M1-7
Before the experiment	75.61	83.36	84.49	95.41	96.77	81.31	93.10
After the experiment	73.78	81.85	83.73	94.08	95.19	82.01	94.35
Mass change (g)	1.83	1.51	0.76	1.33	1.58	−0.7	−1.25

**Table 5:** Composition of concrete mixtures (per m^3^).

Component	M0	M1
Cement (kg/m^3^)	360	360
Silica fume (kg/m^3^)	-	20
Natural aggregates, fraction 0/4 mm (kg/m^3^)	825	750
Natural aggregates, fraction 4/8 mm (kg/m^3^)	235	235
Natural aggregates, fraction 8/16 mm (kg/m^3^)	740	740
Water (L)	170	191
Polycarboxylate plasticizer (L)	3.1	3.1
